# Are Unnecessary Serial Radiographs Being Ordered in Children with Distal Radius Buckle Fractures?

**DOI:** 10.1155/2018/5143639

**Published:** 2018-03-01

**Authors:** Shi-Neng James Ling, Aidan J. Cleary

**Affiliations:** ^1^Department of Orthopaedics, Logan Hospital, Meadowbrook, QLD, Australia; ^2^Department of Orthopaedics, Redlands Hospital, Cleveland, QLD, Australia

## Abstract

*Background. *Torus or buckle distal radius fractures are common injuries in the pediatric population. By definition, they are stable and can be treated conservatively with a wrist splint or soft crepe bandage.* Objective. *The objective of this study was to evaluate the utility of serial radiographs in the clinical outcome of children with stable distal radius buckle fractures.* Materials and Methods. *A one-month retrospective analysis was undertaken at two major hospitals in Queensland—Logan and Redlands Hospital. Statistical analysis was performed to identify any relationships between serial radiographs and certain demographic parameters including fracture characteristics, age, sex, and limb side.* Results. *Of the 136 patients, 50% had more than one radiograph series taken. A total of 576 single radiographs and 251 radiograph series were taken. All fractures healed without complications and did not require active intervention. There was a statistically significant relationship (*p* = 0.0015) between fracture angulation and multiple radiographs series. A cost analysis revealed $55,890 per year could be saved by not performing serial radiographs.* Conclusion. *Serial radiographs did not appear to change the excellent clinical outcome for children with distal radius buckle fractures. There is a potential to reduce costs and prevent unnecessary ionizing radiation exposure to children.

## 1. Introduction

Pediatric patients with a distal radius buckle or torus fracture are a common presentation to hospitals [1]. A torus or buckle fracture is defined as a compression failure of the bone, usually at the metaphyseal and diaphyseal junction of the distal radius. They are undisplaced but may be angulated [2]. These injuries have little to no tendency to displace and thus do not require active intervention. Most standard guidelines recommend treatment with immobilisation in a cast, splint, or soft crepe bandage through their primary care physician [3–7]. Currently, there is no consensus on the timing and frequency of radiographs in the follow-up of these injuries in Australian Hospitals. As a result, unnecessary radiographs may be ordered, exposing children to potentially damaging levels of ionizing radiation [8]. Reducing radiographs would also reduce costs and resources for the health system. Thus, the aim of this study was to assess the utility and frequency of serial radiographs in the management of stable pediatric buckle distal radius fractures.

## 2. Materials and Method

**Figure 1:**
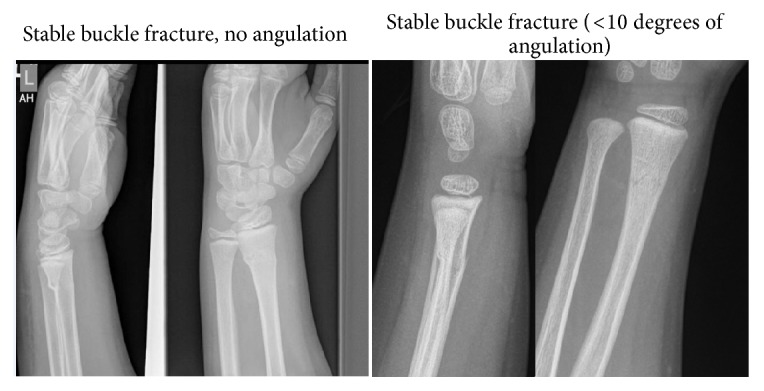
Angulated and nonangulated buckle fractures.

Ethics approval for the study was obtained from Metro South Ethics Committee, approval number HREC/16/QPAH/543. A retrospective, multicentre review of all stable buckle distal radius fractures in patients aged 0–16 was undertaken. All cases which presented to the Logan and Redlands Hospital, Queensland, Australia, over a one-month period from the 1st of February 2016 to the 1st of March 2016 were identified. Both Logan and Redlands Hospital are two major public metropolitan hospitals accepting all pediatric traumas [9]. All cases of distal radius fractures presenting to the emergency department were identified through the hospital's coding system. Computed radiographs were used in both locations. Both authors individually reviewed all radiographs to identify patients who had a buckle fracture that was undisplaced and either not angulated or angulated less than 10 degrees (see Figure 1).

To ensure diagnostic accuracy, the radiologist report was concurrently reviewed with no discrepancies found. Data was collected from the clinical records of all patients with regard to their demographics, type of fracture, clinical outcome, and number of radiographs and radiograph series. “Radiographic series” was defined as an individual episode of radiograph images taken, accompanied by an official request form completed by a medical practitioner. It usually consists of at least two views including anterior-posterior (AP) projection and a lateral projection and occasionally an oblique view. The information was recorded on an electronic spreadsheet. Cases were excluded if they did not meet the definition of a stable buckle fracutre or had images not loaded onto the hospital imaging system or if the clinical record was incomplete. An analysis was performed using Stata 12 (Statacorp, Texas) to identify any relationships between different variables. The chi-squared and Fisher's exact test were used for correlation between categorical variables and the *t*-test was for correlation between continuous variables. A *p* value of <0.05 was considered statistically significant with a 95% confidence interval applied. A cost analysis was also performed using information from the Logan Hospital Radiology Department and the Australian Government Medicare Benefits Schedule (MBS).

## 3. Results

One hundred and thirty-six patients with a buckle or torus fracture were included in the study whose demographics are shown in Table 1.

Radiograph demographics are shown in Table 2. Half of the patients received multiple radiograph series during their treatment course.

All of the 136 fractures were clinically healed as per the medical record without the need for any fracture reduction or active intervention. No re-presentations or complications were identified within at least a 6-month time frame.

An analysis was performed to identify factors which were associated with a patient receiving multiple radiograph series (Table 3). There was a statistically significant association between fracture angulation and a patient having multiple radiograph series (*p* = 0.0021). There were no significant associations with age, limb, side, and gender.

An extra 115 radiograph series were ordered on top of the 136 series that were performed for initial diagnosis. The Australian Medicare Benefits Schedule of 2014 reports a fee of $40.50 for wrist radiographs (15). This equates to a saving of $4657.50 over the audit period, which translates to a potential cost saving of approximately $55,890 between the two hospitals per year.

## 4. Discussion

There are currently no national guidelines for the use of radiographs in managing pediatric buckle fractures. In our study, all patients healed without complication, regardless of whether follow-up radiographs were ordered. This excellent healing rate has been confirmed in multiple studies with patients almost always returning to a full level of function with no ongoing deficit [10–12]. Performing serial radiographs for these injuries thus appear to be unnecessary and did not change clinical management.

We acknowledge that there may be some perceived benefits for ordering serial radiographs for buckle fractures. With medico-legal issues becoming more intertwined in the clinical management of patients, confirming radiological union may provide added protection for the clinician. This information would also be useful when communicating with the child's guardian or carer, which can be difficult at the best of times.

Our analysis identified that fracture angulation was associated with a higher likelihood of repeat radiographic series. The hypothesised causes of this effect are likely multifactorial. We believe that there is an innate attitude of the treating clinicians to continue to monitor fractures that are not perfectly anatomical, despite the evidence clearly showing that the natural history of this type of fracture is the same as that of a nonangulated fracture. This may represent a lack of adequate education for medical staff and an area that could be easily addressed.

It was interesting to note that there was a correlation, however not statistically significant, between the age of a patient and the likelihood of serial radiograph series. Age may play a role in the number of radiograph series performed for multiple reasons. There is an increased potential of remodelling for fractures at younger ages. Also, parent expectations may be different for younger patients as opposed to older patients and this may influence the doctor's decision to perform serial radiographs.

The ALARA or “as low as reasonably achievable” principle is designed to minimize radiation exposure. Particularly in the pediatric population, it is important to attempt to reduce exposure as they are more vulnerable to the effects of ionizing radiation [8]. A child is exposed to an estimated radiation dose of 0.05–0.005 mSv per single limb radiograph [13]. Although this dose is almost negligible in increasing the risk of cancer, the principle of minimizing radiation exposure remains important. This study has found practical and simple ways to achieve this. Further, the potential cost savings of limiting serial radiographs is significant. In a budget conscious health system, the $55,890 per year could be spent in other areas of need.

This retrospective study was limited by its small sample size. Other limitations included not knowing the full indications for why the clinician ordered the serial radiograph. Further qualitative research could focus on the reasons behind this, in an attempt to change clinical practice in order to reduce unnecessary radiographs.

## 5. Conclusion

In our cohort of patients, buckle fractures angulated less than 10 degrees went on to heal without active medical intervention. This indicates that follow-up radiographs may be unnecessary in the clinical management of these injuries. In our practice, we only perform a follow-up radiograph if the patient has clinical evidence of delayed union or a gross anatomical deformity.

## Figures and Tables

**Table 1:** Patient demographics.

Total number of patients	136
Mean age (years)	9
Age range	0–16
Sex (male/female)	83/53
Side (left/right)	83/53
Nonangulated/angulated	95/41

**Table 2:** Radiograph demographics.

Total wrist radiographs	Mean number of radiographs/patient	Patients with single radiograph series	Patients with multiple radiograph series	Total number of radiograph series	Mean number of radiograph series/patient
576	4.24	68	68	251	1.85

**Table 3:** Correlation of factors related to a patient receiving multiple radiograph series.

Factor	Single series	Multiple series	*p* value
Side			
Left	39	44	*p* = 0.3793
Right	29	24	
Sex			
Male	38	45	*p* = 0.2184
Female	30	23	
Age			
Six and under	23	14	*p* = 0.0829
Over 6	45	54	
Displacement			
No angulation	56	39	*p* = 0.0015
Angulated	12	29	
